# Edible and medicinal termites: a global overview

**DOI:** 10.1186/s13002-015-0016-4

**Published:** 2015-04-30

**Authors:** Rozzanna Esther Cavalcanti Reis de Figueirêdo, Alexandre Vasconcellos, Iamara Silva Policarpo, Rômulo Romeu Nóbrega Alves

**Affiliations:** Departamento de Sistemática e Ecologia, CCEN, Universidade Federal da Paraíba, Laboratório de Termitologia, 58051-900 João Pessoa PB, Brazil; Departamento de Biologia, Universidade Estadual da Paraíba, Laboratório de Termitologia, 58051-900 João Pessoa PB, Brazil

**Keywords:** Ethnoentomology, Ethnozoology, Entomophagy, Entomotherapy

## Abstract

Termites are mainly known for damage caused to human beings, both in urban and rural areas. However, these insects play an important role in the decomposition of organic matter in tropical regions and are important natural resources, which are widely used in traditional medicine and are also consumed by human populations in several parts of the world. This study aimed to catalogue termite species used worldwide through a literature review, characterizing them by its human populations’ use. The results showed that at least 45 species of termites, belonging to four families, are used in the world, with 43 species used in human diet and/or in livestock feeding. Nine termite species are used as a therapeutic resource. There is an overlapping use of seven species. The use of termites was registered in 29 countries over three continents. Africa is the continent with the highest number of records, followed by America and Asia. The results suggest that, in addition to their ecological importance, termites are a source of medicinal and food resources to various human populations in various locations of the world, showing their potential for being used as an alternative protein source in human or livestock diets, as well as a source for new medicines.

## Introduction

Since ancient times, complex interactions among humans and other animals have been recorded, including harmonic and conflicting relations [1,2]. Termites illustrate this situation, as they can cause significant economic damage in urban and rural areas. At the same time, people from different parts of the world use them as food (for humans and livestock), and as a source material for popular medicine.

The importance of insects as a food source for humans is not surprising, since this is the group with the highest number of species in nature, thereby representing a significant biomass [3]. Considered as important natural resources, insects are, in many ways, a basic component of the diets of humans and other animals [4] and have played an important role as a source of medicinal resources [5-8]. Entomophagy, as the practice of using insects as a part of the human diet is called [9], has played an important role in the history of human nutrition in Africa, Asia and Latin America [10]. Another important use of insects by humans is medicinal use, featuring a practice known as entomotherapy [8,11].

The negative view that most people have of termites prevails in many places, and it often masks the ecological role of these insects as mediators of the process of decomposition of plant organic matter and as agents with influence on the formation of soils and energy and nutrient flows, especially in tropical forests [12,13]. It should be emphasized that, from a utilitarian perspective, termites are commonly used insects in traditional popular medicine [6,14-16]. They are used in the treatment of various diseases that affect humans, such as influenza, asthma, bronchitis, whooping cough, sinusitis, tonsillitis and hoarseness [17,18]. Additionally, these animals have historically been an important source of food that may contribute to improving human diet, particularly for people who suffer from malnutrition due to a deficit of protein, as they are considered a non-conventional food with great economic and social importance. They have been consumed for generations in many regions of the world, a practice that has increased in popularity in recent years [19-24].

Despite their nutritional and medicinal importance to humans and livestock, termites are most recognized for their role as a plague, with a small widespread utility role. Therefore, the present review aims to catalogue the species of termites used worldwide, compiling the uses of these insects by human populations and delimiting their geographical spread. The research was mainly focused on the following questions: How many species of termites are used by human populations in the world? What are the uses of termite species? Which countries have the use of termite species?

## Methods

To obtain data, available references that recorded the use of termites in the human and livestock diet were analysed, as well as the use of the species in traditional medicine. Information was collected from the analysis of published articles, books and book chapters available in online international databases such as ScienceDirect, CAPES Journals, SpringerLink, Google Scholar, Scopus and Web of Science and on scientific journal websites. The research in databases included the following keywords: termite + food + entomophagy – edible insects + termite – entomotherapy + termite – traditional medicine + termite.

Only identified species were compiled, excluding animals mentioned by their vernacular names or with only genus identification. The following references were consulted [4,6,11,14-16,18,20,23,25-103]. A database was created containing information about termite species, family names and the countries where their use was recorded. Taxonomic information about species has been updated, according to the Treatise on the Isoptera of the World [104].

## Results

A total of 45 termite species belonging to four families were recorded as being used by human populations, with 43 species used in the human diet or for livestock feeding (Table 1) and nine species used as a therapeutic resource (Table 2). It can be seen that seven species can be used for both recorded purposes. Families reported in the studies were Hodotermitidae (2 spp.; 4%), Kalotermitidae (2 spp.; 4%), Rhinotermitidae (7 spp.; 6%) and Termitidae (39 spp.; 87%). The species most frequently recorded in the studies were *Macrotermes bellicosus* (Smeathman, 1781) [n = 22 studies], *Macrotermes subhyalinus* (Rambur, 1842) [n = 20 studies], *Nasutitermes macrocephalus* (Silvestri, 1903) [n = 10 studies] and *Pseudacanthotermes spiniger* (Sjoestedt, 1900) [n = 10 studies]. It should be noted that termite species richness used by humans must exceed the number registered here, especially given that, in many studies, termites are mentioned only by their common name, or their identification is suitable only to genera.

**Table 1:** **Termite species used as food or feed**

**Species**	**Use**		**Country(ies)**	**Reference(s)**
	**Feed**	**Food**		
**Hodotermitidae**				
*Hodotermes mossambicus* (Hagen, 1853)		X	Botswana	[54]
*Microhodotermes viator* (Latreille, 1804)		X	South Africa	[38]
**Kalotermitidae**				
*Kalotermes flavicollis* (Fabricius, 1793)	X	X	Brazil, Thailand	[25,38,51,59]
**Rhinotermitidae**				
*Coptotermes formosanus* (Shiraki, 1909)		X	China	[25,38,57,98]
*Reticulitermes flavipes* (Kollar, 1837)		X	Thailand	[20,25,38]
*Reticulitermes tibialis* (Banks in Banks & Snyder, 1920)		X	Mexico	[38]
**Termitidae**				
*Cubitermes atrox* (Smeathman, 1781)		X	Indonesia	[25,38]
*Labiotermes labralis* (Holmgren, 1906)		X	Colombia	[25,53]
*Macrotermes acrocephalus* (Ping, 1985)		X	China	[25,38,57]
*Macrotermes annandalei* (Silvestri, 1914)		X	China	[25,57,58,98]
*Macrotermes barneyi* (Light, 1924)		X	China	[25,38,57,58,98]
*Macrotermes bellicosus* (Smeathman, 1781)	X	X	Central African Republic, Congo, Democratic Republic of the Congo, Nigeria, Angola, Zambia, Kenya, Guinea, Senegal, Tanzania, Uganda	[4,20,23,25-28,33,34,38,42-47,49,75,80,81,88,96,103]
*Macrotermes falciger* (Gerstacker, 1891)		X	Zimbabwe, Congo, Benin, Zambia, South Africa	[20,25,38-40,48,49,54,77,103]
*Macrotermes gabonensis* (Sjoestedt, 1900)		X	Congo	[25,38]
*Macrotermes gilvus* (Hagen, 1858)		X	Thailand, Malaysa	[25,55,56]
*Macrotermes herus* (Sjoestedt, 1914)	X		Tanzania	[35,79]
*Macrotermes lilljeborgi* (Sjoestedt, 1896)	X		Cameroon, Guinea	[83,84,88]
*Macrotermes michaelseni* (Sjoestedt, 1914)		X	Malawi	[103]
*Macrotermes muelleri* (Sjoestedt, 1898)	X		Congo, Cameroon, Guinea	[36,37,83]
*Macrotermes natalensis* (Haviland, 1898)		X	Central African Republic, Zimbabwe, Congo, Nigeria	[20,25,27,32,38,41,49,95]
*Macrotermes nobilis* (Sjoestedt, 1900)	X		Congo, Gabon, Cameroon	[36,37,83]
*Macrotermes renouxi* (Rouland, 1993)	X		Cameroon	[88,113]
*Macrotermes subhyalinus* (Rambur, 1842)	X	X	Angola, Zambia, Kenya, Senegal, Tanzania, Uganda	[23,25,28-31,34,38,39,43,56,58,76,78,79,88,92-94,103]
*Macrotermes vitrialatus* (Sjoestedt, 1899)		X	Zambia	[25,30,31,38]
*Microcerotermes dubius* (Haviland, 1898)		X	Malaysia	[25,56]
*Microcerotermes serrula* (Holmgren, 1912a)		X	Malaysia	[25,56]
*Nasutitermes corniger* (Motschulsky, 1855)		X	Venezuela	[25,53]
*Nasutitermes ephratae* (Holmgren, 1910b)		X	Venezuela	[25,53]
*Nasutitermes macrocephalus* (Silvestri, 1903)		X	Venezuela	[25,53]
*Nasutitermes surinamensis* (Holmgren, 1910b)		X	Venezuela	[25,53]
*Odontotermes badius* (Haviland, 1898)		X	South Africa, Zambia, Kenya	[25,30,38,47,51]
*Odontotermes capensis* (De Geer, 1778)		X	South Africa	[25,38]
*Odontotermes feae* (Wasmann, 1896)		X	India	[25,38,57]
*Odontotermes formosanus* (Shiraki, 1909)		X	India, China	[25,58,59,100,103]
*Odontotermes kibarensis* (Fuller, 1923)		X	Uganda	[103]
*Odontotermes yunnanensis* (Tsai & Chen, 1963)		X	China	[25,58,59,100]
*Pseudacanthotermes militaris* (Hagen, 1858)	X	X	Angola, Kenya, Tanzania, Uganda	[25,30,44,77,88,90,103]
*Pseudacanthotermes spiniger* (Sjoestedt, 1900)	X	X	Congo, Zambia, Tanzania, Kenya, Uganda	[25,30,38,44,47,77,87,90,96,103]
*Syntermes aculeosus* (Emerson, 1945)		X	Venezuela, Brazil	[25,54,93]
*Syntermes parallelus* (Silvestri, 1923)		X	Colombia	[25,38,57,91,92]
*Syntermes spinosus* (Latreille, 1804)		X	Brazil, Colombia, Venezuela	[4,25,38,54,57,91,92]
*Syntermes tanygnathus* (Constantino, 1995)		X	Colombia	[25,53]
*Termes fatalis* (Linnaeus, 1758)		X	Guyana, Indonesia	[25,38]

**Table 2:** **Termite species used in traditional folk medicine**

**Termite species/family**	**Use**		**Country(ies)**	**Reference(s)**
	**Treated diseases**	**Several**		
**Hodotermitidae**				
*Hodotermes mossambicus* (Hagen, 1853)	Child malnutrition		Zambia	[62]
**Termitidae**				
*Macrotermes bellicosus* (Smeathman, 1781)	Suture wounds		Somalia	[101]
*Macrotermes nigeriensis* (Sjoestedt, 1911e)	Sickness in pregmant women, Wounds	Spiritual protection against witches and wizards, rituals protection and promotion (in jobs, trade), appealing to gods and witches, safe delivery of baby, soothsaying (Afose)	Nigeria	[18,61]
*Microcerotermes exiguus* (Hagen, 1858)	Asthma, Bronchitis, Influenza, Whooping Cough, Flu		Brazil	[6,11,18,61,72,75,99]
*Nasutitermes corniger* (Motschulsky, 1855)	Asthma, Cough, Flu, and sore throat, Antibiotic activity, Antimicrobial	Chickens’ gogo (infectious coryza, a type of cold)/ Honey	Brazil	[15,18,66,68-70,73]
*Nasutitermes macrocephalus* (Silvestri, 1903)	Asthma, Leakage, Bronchitis, ‘catarrh in the chest’ coughs, influenza, sore throat, sinusitis, tonsillitis and hoarseness		Brazil	[6,16,18,65,67,71,73-75,100]
*Odontotermes feae* (Wasmann, 1896)	Asthma		India	[20]
*Odontotermes formosanus* (Shiraki, 1909)	Ulcer, Better health, Body pain, Rheumatics, Anemia, The Enhacement of lactation		India	[14,20,70,102]
*Pseudacanthotermes spiniger* (Sjoestedt, 1900)	Antifungal and antibacterial properties		Brazil	[15,20,63,64]

Among the species used in human and/or livestock diet, the species *Macrotermes bellicosus* stood out, with a record of usage in several countries, especially in Africa. In addition, four species are used both in the human diet and for livestock feeding. Furthermore, five species had only one registered use, including *Macrotermes herus* (Sjoestedt, 1914), *Macrotermes lilljeborgi* (Sjoestedt, 1896) and *Macrotermes muelleri* (Sjoestedt, 1898), which are used only for livestock feeding.

For medicinal purposes, the use of ten species of termites was recorded. These species are used as an alternative treatment for physiological and spiritual problems. The species *Nasutitermes macrocephalus* was the most frequently recorded, and it is widely used in Brazil as a therapeutic resource for the treatment of asthma, hoarseness and sinusitis, among other diseases. Another example is *Macrotermes nigeriensis*, which is used in Nigeria in the treatment of wounds, sickness of pregnant women and as a charm for spiritual protection.

The use of termites was registered in 29 countries over three continents. Africa is the continent with the highest number of records (19 countries), followed by America (5 countries) and Asia (5 countries). Congo recorded the highest number of species used as food by humans and other animals (7 species recorded in the compiled studies), followed by China (6 spp.), Venezuela (6 spp.) and Zambia (5 spp.). Among species with a therapeutic use, there was a predominance of records in Brazil, suggesting that the use of termites in Brazilian popular medicine is relatively common. This kind of use was also recorded in other countries such as India (2 spp.), Zambia (1 sp.) and Nigeria (1 sp.).

## Discussion

The information compiled in the present review shows that the interaction between the people and termites, throughout the world, has not been limited to conflicting relations, especially those related to their role as pests. The significant number of termite species used by people reflects the importance of these animals in different parts of the world, either for food or as a therapeutic resource, which are the main uses of these animals by human populations. Medicinal uses of termites in traditional systems of medicine are indicative that they may have potential as medicinal resources that deserve to be investigated from a pharmacological perspective. Similarly, their use as a protein source reveals that these insects may play important roles in human and other animals’ diets, reinforcing a tendency indicated in previous studies [8,14,16,57,76,77,91,105].

The high number of termites used as food corroborates previous studies that indicated that these animals have been widely consumed by people from all over the world because they provide relevant quantities of nutrients [23,49]. These animals are among the most commonly consumed insects on the planet, second only to grasshoppers [106]. In a survey on the consumption of termites held in Côte d’Ivoire, from 500 people surveyed, 97% consumed or had consumed termites, demonstrating that such use is part of the reality of rural and urban populations in that country, showing a consumption driven by the nutritional value, flavour and aroma of these insects, as well as by the curiosity of the people who consume them [22]. It is noticeable that some species are widely consumed in some countries, suggesting a preference for them, especially those belonging to the genus *Macrotermes*. This predilection, according to Mitsuhashi [107], may be related to differences in the nutritional composition of the genus *Macrotermes*, since they have high levels of proteins and lipids. This may explain why most of the compiled species (37%) in our review belong to this genus. In Africa, this genus is abundant. They are known as “big termites”, and are considered one of the favourite foods, not only of humans but also of gorillas and chimpanzees [83,88,105], thus revealing a curious aspect of termite consumption by humans, since they are also consumed by animals phylogenetically close to humans.

Geographically, among the compiled studies, the highest number of termite species used for food compiled in the present review was documented in Africa and South America, corroborating Ramos-Elorduy [108] who also identified the Americas and Africa as the areas with the highest number of insect species consumed as food. Complementing this information, in Asia, there is a large number of insects used as food by people [25]. One should also point out that most ethnobiological studies that reported the use of termites by humans were carried out in countries of Africa and the Americas, a fact that influenced the results in our review. Currently, more attention is given to alternative food resources traditionally used by humans, which, if used or exploited, are likely to be a more sustainable solution to nutrient deficiency among human populations [76]. In addition, as we can see in our review, termites can provide an important contribution in this regard.

The use of termites as a therapeutic resource also revealed an important mode of use of these animals. Evidence of antimicrobial activity of products isolated from these animals has been reported, such as peptides like espinigerine and termicine, isolated from *Pseudocanthotermes spiniger*, which showed antifungal and antibacterial activities [109]. In the compiled studies, a frequency of *Nasutitermes macrocephalus* (Silvestri, 1903) can be seen in the treatment of various diseases. Studies addressing the molecular biology of termites from the genus *Nasutitermes* demonstrated their potential as antimicrobial peptide producers [110,111]. Chaves et al. [112] suggest that *Nasutitermes corniger* and its nest are promising natural products for use in antimicrobial therapy. In a broader perspective, insects has significant potential as a source of medicinally relevant substances [5]. In this context, the use of termites, because of their wide use in traditional medical systems worldwide, indicates that these animals deserve to be further investigated from a pharmacological perspective. The pharmacological activity of termites could contribute to valorize these animals, whose negative aspects associated with the damage they cause to humans are often highlighted.

The compiled results revealed that several species of termites have historically been used by humans for food or medicinal use, showing a facet of human/termite interaction that, most of the time, is little known, and that, in a way, is masked by the negative perception that most people have of these insects. It has been ascertained that the value of termites as a source of food or popular remedies is widespread in many countries of the world, showing that these animals have been historically exploited by humans from a utilitarian perspective, an aspect that, combined with their ecological importance, may contribute to demystifying the negative view associated with these insects.
